# Dengue & COVID-19: A Comparison and the Challenges at Hand

**DOI:** 10.7759/cureus.31877

**Published:** 2022-11-25

**Authors:** Deekshitha Alla, Sai Santhosha Mrudula Alla, Roopeessh Vempati, Heom Bhatt, Qamar Sultana, Siddharth Bhatt, Tahsina Mohsin, Ayesha Siddiqua

**Affiliations:** 1 Department of Medicine, Andhra Medical College, Visakhapatnam, IND; 2 Internal Medicine, Gandhi medical college & Hospital, Hyderabad, IND; 3 Department of Medicine, Dr. M.K.Shah Medical College and Research Centre, Ahmedabad, IND; 4 Department of Medicine, Deccan College of Medical Sciences, Hyderabad, IND; 5 Department of Medicine, Dr.M.K.Shah Medical College and Research Centre, Ahmedabad, IND; 6 Department of Medicine, Tbilisi State Medical University, Tbilisi, GEO; 7 Department of Medicine, Shadan Institute of Medical Sciences, Hyderabad, IND

**Keywords:** interleukins, rt-pcr (real time - reverse transcription polymerase chain reaction), antibody-dependent enhancement (ade), dengue virus infection, covid-19

## Abstract

The COVID-19 pandemic caused by SARS-CoV-2 spread across many countries between 2020 and 2022. The similarities in clinical presentation with other endemic diseases pose a challenge to physicians in effectively diagnosing and treating the infection. Approximately 129 nations have a risk of dengue infection, and more than 100 of those are endemic to dengue. During the COVID-19 pandemic, the number of dengue cases decreased in many countries owing to the isolation measures followed. However, the common clinical presentation between them has led to misdiagnosis. Both COVID-19 and dengue fever cause a surge in pro-inflammatory cytokines and chemokines, thus sharing a common pathophysiology. False positive serological test results also posed difficulty differentiating between COVID-19 and dengue fever. This review aims to compare the clinical features, pathophysiology, and immune response between dengue and COVID-19, to benefit public health management during the pandemic.

## Introduction and background

The coronavirus disease of 2019 was first identified in the city of Wuhan, China during the latter half of November 2019. Patients presented with fever, cough, anosmia, headache, diarrhea, and skin rash symptoms. On 30th January 2020, the WHO declared this a public health emergency of international concern; on 11th march 2020, it was declared a pandemic. As of 11th October 2022, there have been 619,161,228 confirmed cases and 6,537,636 reported deaths. In an attempt to further control this disease, vaccines were manufactured and a total of 12,723,216,322 vaccine doses were administered globally by 3rd October 2022 [1].

**Figure 1:**
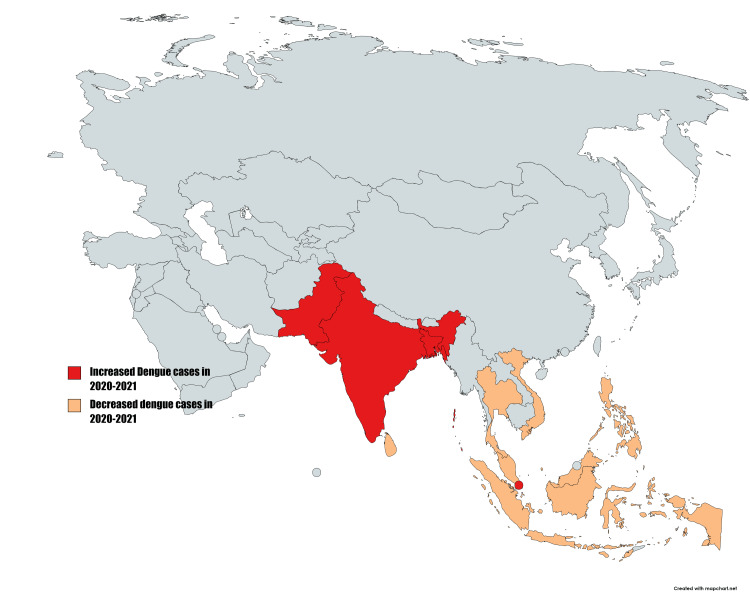
Comparison of dengue cases in dengue-epidemic countries during the COVID-19 pandemic

Dengue is a vector-borne disease caused by the dengue virus (DENV 1-4), a positive sense single-strand RNA virus of the family *Flaviviridae*. It is transmitted by the bite of an infected *Aedes aegypti* mosquito, and to a lesser extent by *Aedes albopictus*, *Aedes polynesiensis*, and *Aedes scutellaris*. Some of the common symptoms include severe headache, retro-orbital pain, myalgia, arthralgia, nausea, vomiting, and rash. Dengue is vastly common in tropical countries and endemic in almost 120 countries with local variations in cases and severity depending on environmental and social factors. Asia contributes to 70% of the global dengue burden. The coronavirus disease of 2019 had a significant impact on dengue cases worldwide. According to the WHO, the total number of dengue cases reported in 2019 was 5.2 million. However, total cases seemingly decreased in 2020 and 2021 [2]. Three Asian countries (Bangladesh, Pakistan, and India) and seven non-Asian countries (Brazil, Peru, Bolivia, Ecuador, Paraguay, Argentina, and Singapore) have reported an increase in the number of dengue cases during the pandemic (Figure 1) [3]. However, an overall reduction in the number of dengue cases in the world indicates a strong association between COVID-19-related movement restrictions and reduced dengue risk. These findings add to the growing body of evidence that dengue is spread through human movement, with transmission occurring in shared areas outside the home through mosquitoes [4]. This review compares the clinical features, pathophysiology, immune response, and the possibility of misdiagnosis between dengue and COVID-19. 

## Review

Methods

By evaluating relevant literature, the current review aims to compare COVID-19 and dengue fever. A literature search was performed in PubMed using the following terms: "dengue and COVID-19" and "COVID-19 and dengue fever symptoms". Articles were reviewed for relevance and included if they contained information about the clinical features, confusion in distinguishing diagnosis, pathophysiology, and immune response in dengue and COVID-19. Papers written in the English language were considered. The selection process resulted in the selection of 942 papers for evaluation, resulting in the final selection of 32 papers that met the inclusion criteria.

Confusion in diagnosis

Dengue fever and COVID-19 share a common clinical presentation. Similar, less common, unique symptoms, etc. are listed in Table 1.

**Table 1:** Clinical comparison between dengue and COVID-19

Clinical characteristic	Dengue	COVID 19
1. Common symptoms	High-grade fever, flushing, myalgia, and headache	Fever, cough, dyspnea, myalgia, headache, sore throat, rhinorrhea, and sputum production (acute phase of illness) Fever, severe dyspnea, tachypnea, decrease in oxygen saturation (<93%), respiratory distress (acute respiratory distress syndrome), stomach pain, bloodshot eyes, diarrhea, dizziness, skin rash, vomiting, sepsis, and shock (hyperactive immune response) [5]
2. Less common symptoms	Severe injury to the liver, kidneys, bone marrow, heart, and brain (expanded dengue syndrome) [6].	Skin manifestations, COVID toes, hoarseness, eye problems, and hair loss [7].
3. Similar symptoms	Fever, dyspnea, headache, cough, and skin manifestations.	Fever, dyspnea, headache, cough, and skin manifestations (multisystem inflammatory syndrome).
4. Unique Symptoms	Retro-orbital eye pain, photophobia, bleeding manifestations, and petechia [8].	Loss of smell leading to taste disturbances, erythematous rashes and urticaria, thrombotic complications, and consumptive coagulopathy [9].
5. Long-term symptoms	Arthralgia, asthenia, generalized malaise (can persist as long as two years after acute illness) [10].	Fatigue, dyspnea, arthro-myalgia, depression, anxiety, memory loss, concentration difficulties, and insomnia (post-COVID syndrome) [11].
6. Symptoms in children	Fever, retro-orbital pain, myalgia, arthralgia, nausea, vomiting, and skin rash (vomiting and rash are more common) [12]	Fever, cough, sore throat, rhinorrhea, headache, nausea, vomiting, and diarrhea (mild illness) [13]
7. Incubation period	5 to 7 days [2]	2 to 14 days [14]
8. Mortality rate	4% in dengue hemorrhagic fever [15]	2% to 7% [14]
9. Way of transmission	Vector-borne transmitted by the bite of Aedes mosquitos [2]	Respiratory droplets. Rarely due to contact with contaminated surfaces.

Dengue outbreaks amidst the COVID-19 pandemic led to misdiagnosis due to similar clinical presentation and also increased the likelihood of co-infection [16]. A case of co-infection was reported by Chowdhury et al. which was initially diagnosed as COVID-19 infection due to a positive RT-PCR test but later confirmed as co-infection based on positive reactivity to IgG and IgM antibodies in the dengue duo test [17]. Another study reported false positive dengue serology in 22% of COVID-19 patients [18]. Studies in Brazil and other regions have reported that both the dengue virus and SARS-COV-2 spread simultaneously as pandemics [19]. Bandeira et al. reported a case that presented with flu-like symptoms and was suspected to have been infected with SARS-COV-2. The patient was started on necessary management but later presented with maculopapular rash, that spread over the neck, chest, and limbs which led to a re-diagnosis as dengue fever. The reverse transcriptase-polymerase chain reaction (RT-PCR) report was positive for SARS-COV-2 and later, the authors concluded that they misdiagnosed the patient to have dengue, and skin rashes were considered complications of COVID-19 [20]. A similar case was also reported by Joob et al. where skin manifestations led to the misdiagnosis of COVID-19 as dengue [21]. Thrombocytopenia and lymphopenia reported in a few COVID-19 cases also add to this confusion [22]. The doubt of possible dengue and COVID-19 co-infection came to light as patients who tested positive for non-structural protein 1 (NS1) antigen and anti-dengue IgM antibody also reported positive COVID-19 rapid antigen test. However, this perception changed after the same patients tested negative in RT-PCR for SARS-COV-2, which discovered the possibility of cross-reactivity in the serological tests of dengue and COVID-19 [16]. Some studies have reported a false positive serological test for COVID-19 in dengue patients and also a false positive serological test for dengue in COVID-19 patients, thus reinforcing the assumption of cross-reactivity [18]. This cross-reactivity also leads to reduced sensitivity of IgM/IgG-based rapid diagnostic tests (RDT) which emphasizes the importance of NS1 antigen-based RDT in dengue diagnosis due to its lack of cross-reactivity with any other viral infection [23].

Overlapping pathophysiology

The COVID-19 virus enters the host cell by binding to the angiotensin-converting enzyme 2 receptors on the cell membrane. The virus entry into the host cell leads to the activation of the inflammatory cascade ultimately causing the release of inflammatory cytokines and inflammation [24]. In contrast, the dengue virus can infect a variety of cell types including endothelial cells. Dengue virus-infected endothelial cells secrete inflammatory mediators, leading to inflammation and plasma leakage [25].

In COVID-19, inadequate and delayed interferon activation has been linked to persistent viremia and the development of severe disease. Patients who advance to develop severe immune dysfunction exhibit a delayed interferon reaction, and a surge in pro-inflammatory cytokines and chemokines such as IL-1-ß, tumor necrosis factor (TNF)-a, C-X-C motif chemokine 10 (CXCL-10), IL-10, IL-18, IL-8, monocyte chemoattractant protein-1 (MCP-1), and macrophage inflammatory protein-1ß (MIP-1ß) [26]. A study found that individuals who developed COVID-19 pneumonia and dengue hemorrhagic fever (DHF) had considerably greater levels of cytokines than those with minor symptoms [26].

Both IL-6 and IL-1-ß increase endothelial permeability thereby causing acute respiratory distress syndrome (ARDS) in COVID-19 patients. This is similar to the mechanism by which plasma leakage, shock, and ascites occur in patients with DHF [27]. The IL-10 hyperactivates the inflammatory process by increasing the proliferation of cluster of differentiation (CD)8+ T Cells which increases the disease severity [28]. This cytokine storm in COVID-19 that leads to plasma leakage and disseminated intravascular coagulation (DIC) is similar to cytokine-induced thrombocytopenia and increased vascular permeability in dengue infection. Host factors like CCL4 and toll-like receptors also play a role in the disease pathogenesis of COVID-19 and dengue hemorrhagic fever [29]. Several studies found that cell adhesion molecules like intercellular adhesion molecule (ICAM)-1 and vascular cell adhesion molecule (VCAM)-1 cause coagulation dysfunction, increased endothelial permeability, and transmigration of monocytes across the endothelium resulting in endothelial inflammation in severe dengue and COVID-19 [30]. The innate immune response causes the blockade of various interferons which are the first line of antiviral defense, leading to the uncontrolled spread of viruses and a worse clinical picture in COVID-19 and dengue fever [31].

**Figure 2:**
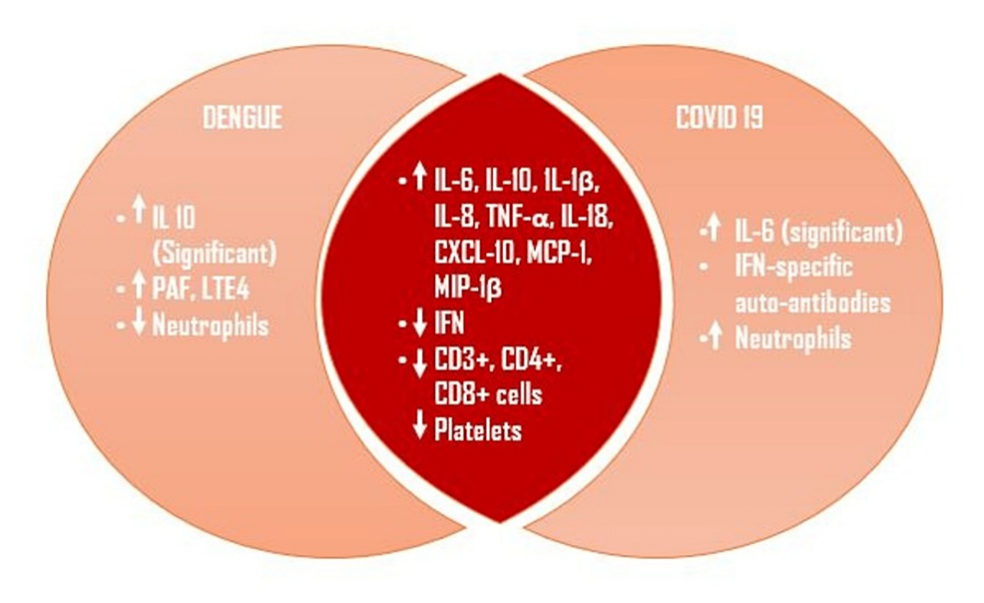
Comparison of innate immune response between dengue and COVID-19 PAF: Platelet activating factor, LTE4: Leukotriene E4, TNF-a: Tumor necrosis factor alpha, CXCL-10: C-X-C motif chemokine 10, MCP-1: Monocyte chemoattractant protein-1, MIP-1ß: Macrophage inflammatory protein-1ß, IFN: Interferon, CD: Cluster of differentiation

By comparing the innate immune response between dengue and COVID-19, lymphopenia (CD3+, CD4+, CD8+) was found in both dengue and COVID-19. Unlike dengue fever, COVID-19 showed a significant increase in neutrophil count (Figure 2) [26].

Humoral immune response in dengue & covid-19

Higher levels of DENV serotype-specific neutralizing antibodies (Nabs) produced by extrafollicular B cell response were proven to grant immunity against re-infection with the same serotype. When a person contracts dengue twice, each time with a different DENV serotype than the first time, the risk of getting DHF is significantly increased due to antibody-dependent enhancement (ADE) by weakly neutralizing and highly cross-reactive antibodies. Unlike dengue, where having a different DENV serotype is a potential risk factor for developing a severe illness due to ADE, this has not been observed with SARS-CoV-2 infection. It has been demonstrated that immunological responses brought on by natural infection have a longer half-life than those brought on by vaccination, and that previous natural infection can prevent the onset of severe clinical disease [26].

**Figure 3:**
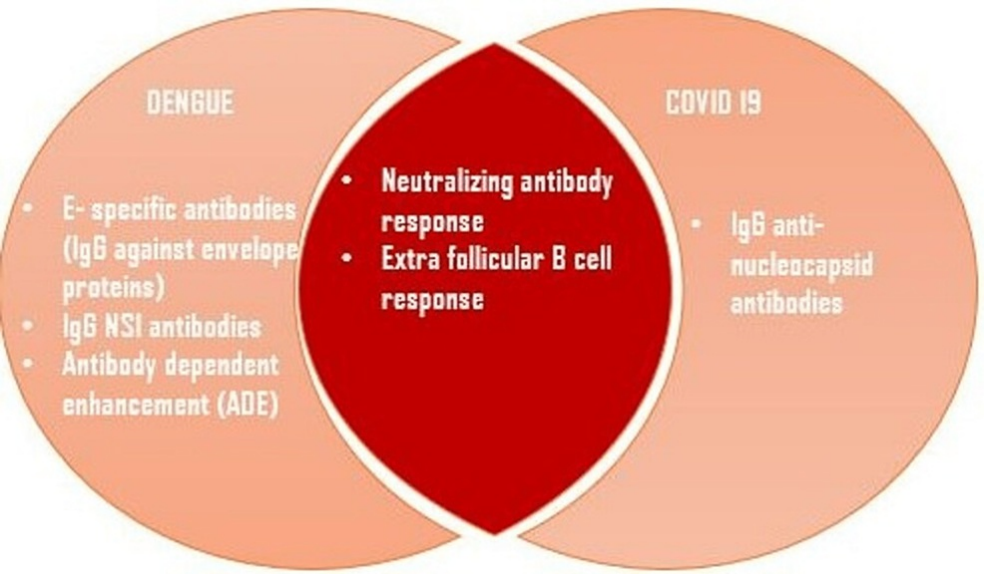
Comparison of humoral immune response between dengue and COVID-19 NS1: Non-structural protein 1

Dengue showed an increase in E-specific antibodies (IgG against envelope proteins), and IgG NS1 antibodies while COVID-19 showed an increase in IgG nucleocapsid antibodies (Figure 3) [26].

## Conclusions

Both COVID-19 and dengue fever pose serious threats to the world. They share similarities in clinical presentations that might lead to confusion in the diagnoses. Common clinical features between dengue fever and COVID-19, such as fever, dyspnea, headache, cough, and skin manifestations lead to confusion. Increased incidence of false positive serological test results due to cross-reactivity and common blood picture also add to this confusion. The misdiagnosis of COVID-19 as dengue and the failure to quarantine such individuals will trigger outbreaks in healthcare facilities. On the other hand, failure to diagnose dengue and administer supportive treatment may lead to preventable dengue-related deaths. Therefore, in areas where dengue and COVID-19 co-exist, patients should be tested for both infections.
